# Techno-Economic Analysis of Producing Glacial Acetic Acid from Poplar Biomass via Bioconversion

**DOI:** 10.3390/molecules25184328

**Published:** 2020-09-21

**Authors:** Rodrigo Morales-Vera, Jordan Crawford, Chang Dou, Renata Bura, Rick Gustafson

**Affiliations:** 1 Center of Biotechnology of Natural Resources (CENBIO), School of Agricultural and Forest Sciences, Catholic University of Maule, 3480112 Talca, Chile; 2 Bechtel Corporation, Reston, VA 20190-5918, USA; jordan.crawford@gmail.com; 3 School of Environmental and Forest Sciences, University of Washington, Seattle, WA 98195-2100, USA; changdou@uw.edu (C.D.); renatab@uw.edu (R.B.); pulp@uw.edu (R.G.)

**Keywords:** acetic acid, techno-economics assessment, poplar, bioconversion, biomass, biorefinery, platform chemicals, organic acids, poplar

## Abstract

Most of the current commercial production of glacial acetic acid (GAA) is by petrochemical routes, primarily methanol carbonylation. GAA is an intermediate in the production of plastics, textiles, dyes, and paints. GAA production from biomass might be an economically viable and sustainable alternative to petroleum-derived routes. Separation of acetic acid from water is a major expense and requires considerable energy. This study evaluates and compares the technical and economic feasibility of GAA production via bioconversion using either ethyl acetate or alamine in diisobutylkerosene (DIBK) as organic solvents for purification. Models of a GAA biorefinery with a production of 120,650 tons/year were simulated in Aspen software. This biorefinery follows the path of pretreatment, enzymatic hydrolysis, acetogen fermentation, and acid purification. Estimated capital costs for different scenarios ranged from USD 186 to 245 million. Recovery of GGA using alamine/DIBK was a more economical process and consumed 64% less energy, due to lower steam demand in the recovery distillation columns. The estimated average minimum selling prices of GGA were USD 756 and 877/ton for alamine/DIBK and ethyl acetate scenarios, respectively. This work establishes a feasible and sustainable approach to produce GGA from poplar biomass via fermentation.

## 1. Introduction

There is considerable interest in biorefining strategies converting biomass into biofuels and platform chemicals. These have been investigated with the goal of reducing greenhouse gas emissions and developing domestic industries, especially those that support rural communities. Platform chemicals refer to chemicals that are commonly used or that are used to synthesize other chemicals. Production of platform chemicals from biomass offers a promising opportunity to reduce U.S. dependence on imported oil, in addition to improving the overall economics and sustainability of integrated biorefineries [1]. Several bio-based chemicals have been proposed as alternatives of petroleum-based chemicals, including organic acids (such as succinic acid [2]), lactic acid [3], acetic acid [4], and itaconic acid [1]; polymers (such as polyethylene) [5], polylactide [6], polyhydroxyalkanote [7], and starch-based polymers [8]); diols [9,10], and sugar-based alcohols (such as sorbitol and xylitol [11]). In general, the downstream product purification cost for fermentation-based processes accounts for more than 60% of the total production cost [2]. In the case of organic acids, such as acetic and succinic acids, industrial separation, and purification from fermentation broths needs further improvement to be economically viable [2,12].

Acetic acid is an important carboxylic acid with a wide range of potential end uses. Acetic acid is used to make products, such as paints, plastics, adhesives, and food. Currently, acetic acid is primarily made by methanol carbonylation [13]. The process works by reacting methanol with carbon monoxide in the presence of a metal carbonyl catalyst (Cativa process) [14]. Carbonylation of methanol to produce acetic acid has a cradle-to-gate global warming potential of 1 kg CO_2_ eq./kg of acetic acid, and manufacturing acetic acid contributed approximately 13.45 million tons of CO_2_ eq. to the atmosphere in 2013 [15]. Production of acetic acid from biomass might be a more sustainable approach to producing this important chemical, provided it is economically viable.

The global demand for acetic acid has steadily increased during the past 10 years. In 2000, global acetic acid demand stood at only 6 million tons, before increasing to 10 million tons in 2011 [16]. In 2014, the global acetic acid market was 12 million tons and is expected to reach 16 million tons by 2020, showing a compound annual growth rate of 4.9%. The Asia-Pacific region is the biggest user of acetic acid, consuming approximately 60% of the total global production in 2014. China is the biggest individual consumer, and is also among the fastest-growing markets with an estimated compound annual growth rate of 5.6% from 2014 to 2020. The European and North American markets are comparatively mature, with demand growth below the average market growth [17]. The bio-acetic acid market is expected to grow at a compound annual rate of above 4% during the next five years. The major factors driving the growth of this market are the rising crude oil prices, increasing demand for the biobased vinyl acetate monomer (VAM) market, and stringent government regulations. The development of new separation technologies to increase production efficiency is likely to provide opportunities for this market [18].

In the last twenty years, several techno-economic (TEA) studies have been performed on biofuels production from biomass [19,20,21,22,23,24,25,26,27,28,29,30,31]; but just recently, a few studies have focused on the economic analysis of a platform chemical production [32,33,34,35]. However, no TEA studies have been published on the production of acetic acid via bioconversion from cellulosic biomass. This work is part of a collaboration between industry, government, and academia to develop a sustainable biofuels and biochemicals industry. Moreover, this is the first study exploring economically purification alternatives of acetic acid. While the specific results presented here apply to acetic acid, the general conclusions will be applicable to the production of any organic acid starting from biomass resources. Importantly, the work shows that for organic acids, downstream separation and recovery processes play a more dominant role in the energy demands and operating costs of the biorefinery; in contrast to bioconversion processes producing fuels, such as ethanol, which are easier to recover from aqueous streams following fermentation.

The goal of this study was to perform techno-economic analyses of glacial acetic acid (GAA) production from poplar wood using two different liquid–liquid extraction (LLE) recovery processes; one with a conventional low boiling point solvent, ethyl acetate; and a second using an organic phase containing a high boiling point tertiary amine, alamine, diluted in kerosene. Separation of acetic acid from water is a major expense and requires considerable energy; consequently, biorefinery performance using different fuels, lignin, and natural gas, was also investigated. Different acetic acid production scenarios are compared through detailed techno-economic analyses of candidate processes. Aspen Plus process simulation software was used to perform mass and energy balances for both ethyl acetate and alamine LLE product recovery scenarios, assuming process steam comes from either combusting lignin or natural gas. The modeled processes used 227,000 tons/year of poplar feedstock, producing an average of 532 kg of acetic acid per bone dry ton of poplar. The scale of an actual factory will depend on many factors, such as biomass availability and cost, and market size. The capacity of the modeled process was chosen as a reasonable compromise between the economies of scale, which favor a very large biorefinery, and the ability to source sufficient biomass feedstock, which increases in cost as the shipping distances increase. Capital and operating expenses are calculated for each configuration, and profitability assessed using discounted cash flow analysis to establish minimum acetic acid selling prices (MSP). Sensitivity analyses are performed to further investigate economic viability.

## 2. Methods

### 2.1. Process Design and Simulations

#### 2.1.1. Feedstock

Short rotation woody crops, such as hybrid poplar, present an attractive option for diversifying and expanding biomass for biofuel production. In the Pacific Northwest region of the United States, there is 20,000 ha hybrid poplar in production [36], with yields ranging from 7 to 19 metric ton ha^−1^year^−1^ [37]. Hybrid poplar costs of 66–110 USD/dry ton were obtained from GreenWood Resources, the leading company in commercial poplar wood production [38]. Poplar exhibits rapid growth in diverse geographical areas [39], requires minimal fertilizer, can be cultivated on marginal lands, has the ability to re-sprout after multiple harvests (coppice), and has high biomass productivity. The lignocellulosic material in poplar wood can be fractionated without extensive pretreatment [40], and hardwoods do not exhibit the recalcitrance reported in softwoods [41]. These characteristics make hybrid poplar an excellent raw material for a lignocellulosic-based biorefinery in this region. The composition by dry mass assumed for the biomass entering the biorefinery, which falls within ranges given in the literature [39,42], is cellulose (42%), hemicellulose (22.9%), and lignin (25.8%). Other minor components of the biomass include extractives (4.5%), acetate (2.9%), and ash (1.9%). It was assumed that the biomass enters the biorefinery with a 50% moisture content.

#### 2.1.2. Feedstock Conversion to Acetic Acid

**Figure 1:**
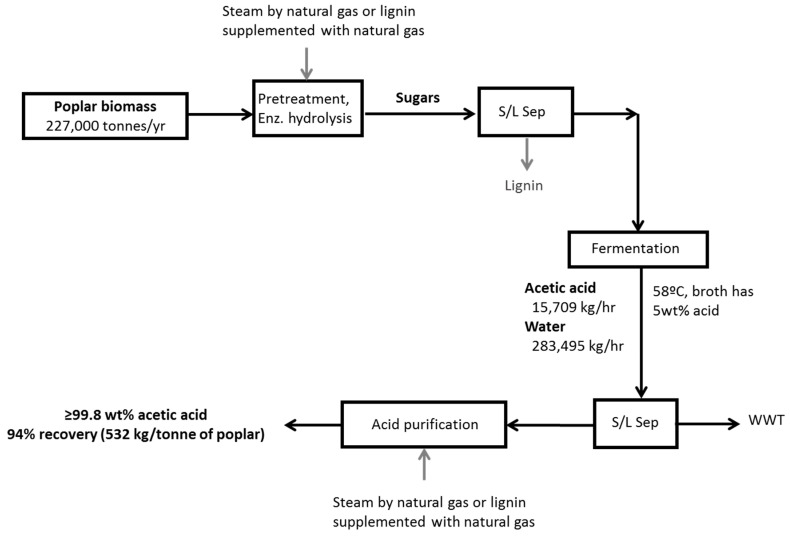
Flow diagram of production of glacial acetic acid (GAA). This biorefinery follows the path of pretreatment, enzymatic hydrolysis, acetogen fermentation, and acid purification. Solid–liquid separation (S/L Sep). Waste Water Treatment (WWT).

Beginning with 27,000 kg/h of chipped hybrid poplar, the first section of the process ends with a dilute fermentation product stream created by fermenting hydrolyzed cellulosic sugars. These process steps are outlined in Figure 1. Most of the steps are commonly modeled [20,21,29,30], with the exception that the fermentation product is usually ethanol instead of acetic acid. In the present study, the process model was run in ASPEN Plus, chemical engineering software, using the NRTL base property method, except for the acetic acid purification, which used NRTL-HOC. Process flows refer to a biorefinery consuming 227,000 bone dry tons per year of biomass. All major reactions were modeled with an RStoic block, and literature values were used for operating temperature and pressure, fractional conversion data, and heat of reaction where applicable. Yields and conversion factors were conservative estimates in collaboration with our research laboratory and industry partners [4,43].

Similar to Crawford et al. (2016), the first two major unit operations, pretreatment, and enzymatic hydrolysis, convert raw cellulosic biomass into fermentable sugars [22]. Dilute acid pretreatment is used, and the stream is then cooled, and pH adjusted for enzymatic hydrolysis where cellulose is converted into fermentable sugars. During pretreatment, a sulfuric acid charge of 0.011 g g^−1^ of dry biomass is used, and the reactor operates at 200 °C. A quantity of 75 wt% of xylan is converted to xylose during pretreatment. Enzymatic hydrolysis occurs at 50 °C, and 91 wt% of cellulose is converted to glucose. Enzymes are assumed to be added at 20 mg g^−1^ cellulose [20] and purchased externally. An acetogenic bacterium, *Moorella thermoacetica*, is employed to ferment both five and six-carbon sugars of the hydrolysate with acetate as the only product [4]. During fermentation, 94 wt% of glucose and 92 wt% of xylose are converted to acetic acid [43]. To maintain an acceptable pH for fermentation, the acetate product is neutralized by the addition of calcium carbonate. A solution of about 5 wt% calcium acetate leaves in the fermentation broth. A final solid–liquid separation takes place to separate the microorganism, which may be performed with a cross-flow filtration system.

#### 2.1.3. Acetic Acid Purification

**Figure 2:**
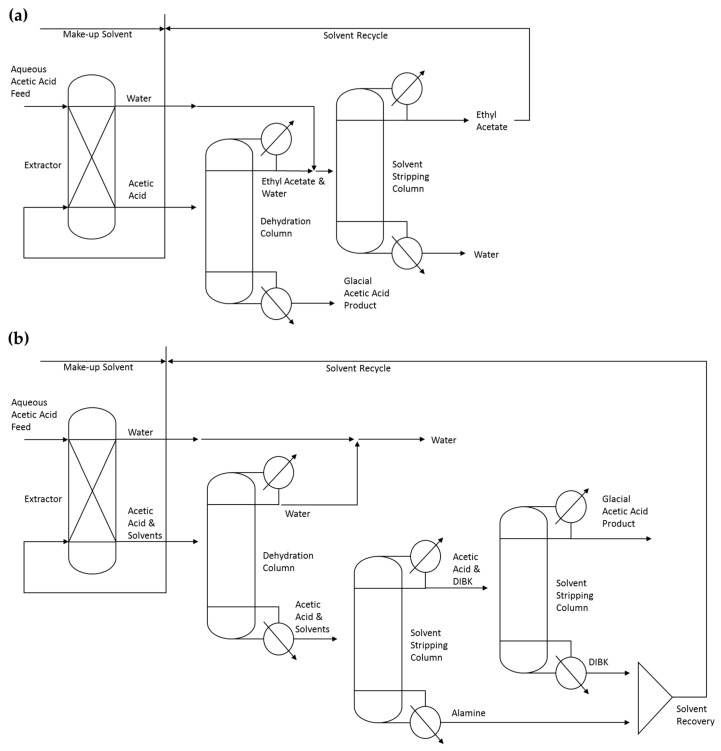
Generalized flow diagrams for the extraction of acetic acid methods investigated: (**a**) Extraction of acetic acid with ethyl acetate; (**b**) extraction of acetic acid with alamine dissolved in diisobutylkerosene (DIBK).

The 5 wt% calcium acetate solution is acidified by the addition of CO_2_, resulting in the formation of calcium carbonate as the calcium acetate is protonated to form acetic acid. The 5 wt% acetic acid fermentation broth is then purified into GAA (99.8 wt% acetic acid). The liquid–liquid extraction unit operation plays a key role in the overall biorefinery. Two different processes are modeled. A more conventional liquid–liquid extraction using ethyl acetate as a solvent (Figure 2a), and a high boiling point solvent extraction using alamine dissolved in kerosene, diisobutylkerosene (DIBK) (Figure 2b). Design specifications within the ASPEN distillation column blocks were tailored to achieve glacial purity (>99.8 wt% acid) and good acid recovery from the solvent phases.

##### Ethyl Acetate Acetic Acid Purification

This area combines three major pieces of equipment, a liquid–liquid extractor and two distillation columns. The dilute acid stream (~5 wt% acetic acid) is mixed with recycled ethyl acetate and a small amount of makeup extractant. The extractor is modeled as an Extract block. The dehydration and stripping columns are modeled as RadFrac blocks. In the dehydration column, the ethyl acetate is boiled overhead, leaving GAA at the bottom of the column. Finally, in the stripping column, ethyl acetate is recovered from the aqueous phase effluent. The aqueous effluent leaving the bottom of the column contains traces of ethyl acetate and acetic acid, and is routed to wastewater treatment (Figure 2a).

##### Alamine/DIBK Acetic Acid Purification

Similar to the ethyl acetate recovery process, the dilute acid stream is mixed with a recycled mixture of equal parts of alamine and DIBK plus a small amount of makeup extractant. This recovery configuration combines four major unit operations: The extractor, which is modeled as an Extract block, and three distillation columns modeled as RadFrac blocks. In the first column, water is boiled overhead, leaving the acetic acid with the solvents at the bottom of the column. The next column separates alamine from acetic acid and DIBK, which are boiled overhead leaving pure alamine in the bottom of the column. In the final column, acetic acid is recovered overhead and the DIBK solvent is left at the bottom of the column (Figure 2b).

#### 2.1.4. Boilers, Electricity Production, and Wastewater Treatment

The boiler simulation, for the processes where lignin is burned with natural gas, is similar to the corn stover to ethanol US National Renewable Energy Lab (NREL) ASPEN model [20] with some variations. An Rstoic reactor is used for the burner, with the option selected for ASPEN to generate combustion reactions. The combustor burns lignin and natural gas. The burner/boiler is modeled to run at 80% efficiency. The boiler is assumed to operate at 6102 kPa. Boiler feed water is preheated and pumped to the operating pressure, where it goes through a heat exchanger (HeatX) with the combustion gases. Steam is generated at 455 °C. When burning lignin and natural gas, lime is added to the flue gas for flue gas desulfurization. The amount of lime is adjusted by ASPEN design specification to enable the complete conversion of flue gas SO_2_ into calcium sulfate. The natural gas boiler is straightforward combustion of methane to make steam for the different processes. Electricity is generated onsite in scenarios in which lignin with natural gas fuels the boiler. High-pressure steam from the boiler goes through a multistage turbine (modeled as separate turbines) with process steam being extracted as needed for pretreatment and liquid–liquid extraction processes. Wastewater treatment is based on the NREL model design [20], but only includes anaerobic and aerobic digesters. The methane-rich biogas from the anaerobic digestion is combusted in the burner for the lignin/natural gas combustion scenario.

### 2.2. Economic Analysis

#### 2.2.1. Equipment and Capital Costs

Equipment and capital costs were estimated using a well-known method of factored estimation, and may include uncertainties of as much as ±30%. The method starts with the costs of major pieces of equipment based on the literature or Capital Cost (CAPCOST) software estimation [20,44]. The NREL (2011) publication was used as a basis for feedstock handling through fermentation, in addition to the wastewater treatment and the burner/boiler system [20]. The cost of the natural gas boiler was obtained by quotes from US vendors. Using the flow data generated during process simulation, the cost of major pieces of equipment was scaled based on Equation (1). The flow components, Flow_a_ and Flow_b_, are simply mass, heat, or work data generated from the simulation. The scaling exponent, n, is found in the literature and is different for various types of equipment, but usually is in the range of 0.5 to 0.8.

(1) $${\mathrm{Cost}}_{a}={\mathrm{Cost}}_{b}\times {\left(\frac{{\mathrm{Flow}}_{a}}{{\mathrm{Flow}}_{b}}\right)}^{n}$$

The scaled cost was then multiplied by an installation factor, and updated to 2018 USD using the Chemical Engineering Plant Cost Index.

The installed equipment comprises the bare module cost, which was then multiplied by a factor of 1.68 to include additional costs: Contingency, contractor fee, site development, auxiliary buildings, off-sites, and utilities [44]. The final estimated capital expense was grassroots, which refers to a completely new facility, although it does not include the cost of land.

#### 2.2.2. Operating Expense (OPEX)

OPEX estimation was done as follows: A list of inputs and outputs of the biorefinery model was compiled. This included raw materials entering the biorefinery, and waste and byproducts exiting the facility. Costs were associated with each item, based on literature sources or publicly available industry quotes. Some costs, such as enzymes, were not narrowly defined, but may be estimated based on the literature. Enzyme costs were 5.22 USD/kg when using 20 mg of enzyme protein per gram of cellulose. Fixed costs, which include labor, maintenance, overhead, administration, and various other support activity costs, were estimated based on Turton et al. (2009) and Humbird et al. (2011) [20,44]. Labor costs were estimated by the number of employees and salaries, while other fixed costs were estimated by factors of labor or capital. Any excess electricity produced in the biorefinery is sold to a nearby grid at USD 0.05 per kWh. Operating costs per hour, year, or volume of acetic acid were estimated based on the simulation results.

#### 2.2.3. Discounted Cash Flow Rate of Return Analysis

One way to measure and compare the profitability of investments, a discounted cash flow rate of return, or internal rate of return analysis, takes into account an entire project timespan. In summary, the analysis manipulates the acetic acid selling price to find the price point, at which the project’s net present value is zero. This calculation is performed by iteration with a specified discount rate of 15%, and the final price is the minimum acetic acid selling price on a weight basis. The 15% discount rate was chosen as a reasonable rate of return to be attractive to investors. The minimum selling price is the minimum price that the acid must be sold for in order to be profitable under the assumed discount rate. Selling the product for a higher price will increase the internal rate of return, while selling for a lower price will reduce the rate of return.

The key parameters of the analysis are shown in Table 1. The minimum selling price uses a pre-tax position (0% tax rate). A pre-tax calculation was chosen because the tax requirement for a given biorefinery is unclear. In addition, because bio-based chemical production is an emerging industry, there is potential for substantial government subsidies and tax breaks.

## 3. Results and Discussion

The results of the Aspen simulations of the various process scenarios, detailed in Section 2, were used to assess technical feasibility and economic viability. The following discussion provides details of the results of the technical and economic analyses.

### 3.1. Process Analysis

Analysis of process simulation provides insights into the technical feasibility of different processes. In most cellulosic to biofuels biorefineries, the generation of heat and power is completely satisfied by the combustion of the lignin fraction of biomass [20,21,29,33]. In this study, recovery of acetic acid from dilute aqueous streams consumes considerable energy; therefore, an external energy source must be considered. We investigated two different energy sources: Natural gas combustion and lignin supplemented with natural gas combustion. The natural gas scenario was considered a lower capital cost approach and used a medium temperature boiler, and did not include a turbo generator to produce electricity. The lignin supplemented with natural gas scenario is similar to the situation in modern pulp mills and—like in a pulp mill—used a high-pressure boiler and high-pressure steam to produce electricity in a turbo generator.

Heat duty, steam, and electricity play a large interconnected role within the biorefinery, especially when different energy and recovery scenarios are compared. Table 2 shows the temperature and heat duty of the different columns during the extraction and purification of acetic acid using the two different solvents. The alamine/diisobutylkerosene (DIBK) columns run at higher temperatures, ranging from 167 to 183 °C, because alamine is a high boiling point extractant. The use of alamine as a solvent, however, results in 64% less energy requirement for acetic acid separation. Ethyl acetate is a more common acetic acid extractant, but because it is more volatile than acetic acid, it must be boiled off from the acetic acid product. When a high boiling point solvent, such as alamine is used, acetic acid is the overhead component. This results in lower energy consumption because the volume of acetic acid is considerably less than that of the extractant [45]. In addition, organic bases provide for substantially higher acetic acid equilibrium distribution coefficients (Kd) than conventional solvents, such as ethyl acetate [46]. Consequently, 29% less solvent was used when the alamine/DIBK mixture was the organic solvent for LLE purification. Wardell and King (1978) confirmed favorable values of Kd (>1) for tertiary amine extractants with an appropriate diluent, such as kerosene [47].

Table 3 summarizes steam usage, temperatures, and pressures in major unit operations. Acetic acid separation and purification, for both scenarios, are the largest steam consumers. Dehydration towers consume the most steam, followed by the separation of the solvents from the final product. Reboiler condensates from these columns may be returned to the boiler, enabling some heat recovery and reducing the overall water usage. The alamine/DIBK process requires an additional stripping tower to separate DIBK from the acetic acid. The configuration of the alamine system and the favorable extraction equilibria of the high boiling point solvent [45] result in steam savings of 68% and 55% for dehydration and stripping processes, respectively, relative to the ethyl acetate system. Dilute acid pretreatment steam demand is the same in both processes. This requires higher pressure steam than the distillation columns, but has a much lower heat duty. Steam used for distillation is returned to the boiler, whereas steam used in pretreatment is directly injected into the process with the water ending up in waste treatment.

Table 4 shows the net biorefinery electricity per kg of acetic acid produced. When lignin and natural gas are burned, 14% and 30% of the electricity generated by the turbogenerator is used in the biorefinery for the ethyl acetate and alamine processes, respectively. Similar to other biorefineries models, the surplus is sold to the grid for credit [20,21,29,33]. This is reflected in economics by an operating cost credit equal to this amount of electricity. When only natural gas is burned, 100% of the electricity is imported from the grid. It should be noted that for the natural gas-based process, our goal was to keep the capital cost to a minimum; hence, we configured the process with a moderate pressure boiler and no steam turbine to produce electricity. In the process of using lignin and natural gas fuels, there is the potential to produce low-carbon electricity from the combustion of the lignin, so the process was configured with a somewhat higher pressure boiler and a steam turbine to provide power for the biorefinery and to sell on the grid.

Net electricity production of this biorefinery is higher compared to those of similar studies that produce ethanol and hydrocarbons instead of GAA [20,21,22,23,24,25,26,27]. Similarly to Vasconcelos (2020) [29], when more steam is required by the process, a larger surplus of electricity is generated by the biorefinery. The greater amount of steam required for the distillation columns also results in more steam being run through the steam turbine producing electricity. 67% more electricity is generated under the ethyl acetate scenario because it requires considerably more steam for distillation than when alamine is used as an extractant. The lower electricity production when alamine is used reduces the income and profitability of the biorefinery and affects the life cycle environmental impacts because of lower electricity exports to the grid [48].

### 3.2. Economic Analysis

Capital costs (CAPEX) were estimated, as described in Section 2. Table 5 shows the costs for a biorefinery facility producing 120,650 tons acetic acid per year broken down into major processes for the different scenarios. Total capital expenses range from USD 186 to 245 million. Identical capital costs were estimated for all of the scenarios for feedstock through fermentation, and wastewater treatment. The alamine/DIBK process, on average, had 7% lower capital requirements than the ethyl acetate process, even though it had an additional column, because it required a smaller steam boiler.

The lower cost scenarios, natural gas only fuel and no electricity production, required 19% and 16% less total investment compared to the lignin/natural gas scenarios when ethyl acetate and alamine were used as extractants, respectively. Natural gas burners are considerably cheaper than those that can use lignin as a fuel [49], and the elimination of the multistage turbine for power production further reduces the cost.

Taking data provided by Smejkal et al. [50], we calculated the 2018 capital cost of a biorefinery producing acetic acid to be 0.5 USD per annual kg of capacity. This calculation was made using the same production as our process, but using methanol carbonylation. The present study estimates the capital cost to be on the order of 1.8 USD per annual kg of acetic acid production. There is an approximately 3.5× difference in the capital costs between the methanol carbonylation-based and cellulosic biomass-based processes. This difference is due to the complexity of the equipment used in the bioconversion processes, which represents around 60% of the total capital cost. A cellulosic-based biorefinery usually is more capital intensive than a non-cellulosic plant. In the case of ethanol, a cellulosic plant is 5–10× more expensive per liter of ethanol than a sugar cane or corn-based ethanol factory [29,30,51,52].

Table 6 shows an operating expense breakdown, in USD, for facilities producing 120,650 tons of acetic acid per year. Feedstock is assumed to be bought at the facility gate, chipped, at 77 USD per bone dry ton (USD 70/ton), and natural gas is priced at 0.004 USD per MJ on a higher heating value basis. Electricity and lignin exported are sold at 0.05 USD per kWh and 81 USD per dry ton, respectively. Electricity must be purchased in the natural gas only case. In the scenario where lignin is burned on-site, supplemented with natural gas, excess electricity is produced and sold. Cash cost is defined here as the production cost to make one ton of acetic acid, not including any discount, tax, capital depreciation, or other factors. It includes only the operating costs, both fixed and variable, and takes into account any credit for revenue generated from selling electricity or lignin as a coproduct. All assumptions for the discounted cash flow analysis to calculate the minimum selling price are discussed in Section 2, where a 15% discount rate was used.

The economic analyses show that feedstock and natural gas cost are the main drivers for the cash production cost of acetic acid, accounting for 23% to 33%, and 18% to 44%, of the total operating cost, respectively. Similar to other biorefinery facilities studies [20,21,22], enzymes are also major contributors to the operating costs, accounting for as much as 17% of the total cost. The “other chemical” prices of the biorefineries burning lignin are somewhat higher than in the natural gas only factories because lime is used for the desulfurization of the flue gas when lignin is combusted.

When lignin is burned on-site a surplus of electricity is sold to the grid for an operating cost credit. Sales of electricity result in a 28% reduction in the cash cost for the ethyl acetate extraction scenario and a 10% reduction in cash cost when alamine is the extractant. Exportation of lignin to a coal-burning facility results in a cash cost reduction of about 8% in the two natural gas only scenarios. Electricity and substantial volumes of natural gas, however, must be purchased in the natural gas only scenarios. These additional utility costs result in a total cash cost that is 20% and 7% greater for the ethyl acetate and alamine scenarios, respectively, in comparison to the cases involving the burning of both lignin and natural gas and generating electricity.

Minimum acetic acid selling prices with a 15% discount rate are estimated to range from 746 to 903 USD/ton for the different scenarios (Table 6). Most of these scenarios are comparable to the high end of the historical market prices in the range of 400 to 850 USD/ton. Ethyl acetate extraction and burning only natural gas shows the highest minimum selling price (903 USD/ton). In this case, the relatively large saving of 60% for the steam plant is not reflected in the final acetic acid selling price, due to the high natural gas price and lack of an electricity production credit.

The minimum selling price for the ethyl acetate biorefineries is much higher than the alamine process. Ethyl acetate requires bigger boilers and uses considerably more fuel. The decision to use the lower capital cost natural gas only process for steam is not clear cut. The low capital cost of the natural gas only scenario is desirable, but the loss of electricity production impacts the final minimum selling price. The optimal biorefinery configuration will be highly dependent on the cost of building the biorefinery (including the need to borrow capital funds), and the price of the fuel and electricity in the region in which the biorefinery operates.

The capital cost of the boiler and fuels, even with the low energy alamine process, are major expenses to produce acetic acid. One approach that could substantially mitigate these costs would be to co-locate the biorefinery with a power plant where we could take advantage of an abundance of medium- and low-pressure steam, which are typically not large sources of revenue in electricity generation. Another alternative is the integration of this second generation cellulosic biorefinery (2G) to a first-generation sugar-based facility (1G), due to the possibility of sharing the existing infrastructure and increasing the potential for energy optimization [29,53,54,55].

### 3.3. Sensitivity Analysis

The previously shown results are a case study using average values for costs and prices. Many economic variables affect the biorefinery finances, however, and these can be quite volatile. The natural gas price is one of the main drivers of acetic acid production operating cost, and its price fluctuation in the US has been substantial [56]. A brief sensitivity analysis was conducted to study the impact of natural gas price on the minimum acetic acid selling price for all of the scenarios. The natural gas price during June 2008 of 12.0 USD/GJ and the average price of 2016 of 2.4 USD/GJ were chosen to perform the analysis [57].

Table 7, compares the minimum selling price of the base case scenario assuming a natural price of 4.2 USD/GJ, and the minimum acetic acid selling price for two projected cases, which assumed a natural gas of USD 2.4 and 12.7 per GJ, respectively. It can be seen in Table 7 that lowering the natural gas price by USD 1.8 per GJ results in a reduction of the minimum acetic acid selling price, on average, of USD 104 per ton for the ethyl acetate process and USD 45 per ton for the alamine process. The significant decrease in minimum acetic acid selling price with a historic reduction in the natural gas price reflects how this critical driver of the operating cost impacts the overall process economics.

Similarly, when there is a significant increase in the natural gas price, as there was in 2008, the minimum selling price of acetic acid from poplar increases by, on average, 52% and 25% for the ethyl acetate and alamine processes, respectively. None of the bio-based acetic acid processes were price competitive, even with historically high acetic acid prices, at these high natural gas prices. However, it should be noted that higher natural gas prices will also significantly drive up production costs using methanol carbonylation. Natural gas prices in the US can be volatile. Biorefineries may need to use strategies, such as long-term contracts or natural gas futures, to mitigate this volatility and to remain profitable.

#### Feedstock and Enzymes Cost

Similar to other bioconversion based biorefineries, biomass, and enzymes cost are other significant contributors to the production cost of acetic acid produced from poplar [20,21,29]. A sensitivity analysis was performed to determine the impact of biomass and enzyme price on the acetic acid minimum selling price. The acetic acid cost was calculated from process simulations by varying the feedstock from 66 to 110 USD/metric ton for each scenario. This range of feedstock cost was supplied by GreenWood Resources [38]. The results of the sensitivity analysis are shown in Figure 1. The minimum selling price of acetic acid ranges from USD 726 to 944 per ton of acetic acid for all the projected cases and scenarios. Reducing the biomass cost from the base case of USD 77 per ton to USD 66 per ton reduced the average operating costs, for all the scenarios, by approximately 4% per ton, translating into a reduction of the minimum acetic acid selling price of about USD 21/ton.

**Figure 3:**
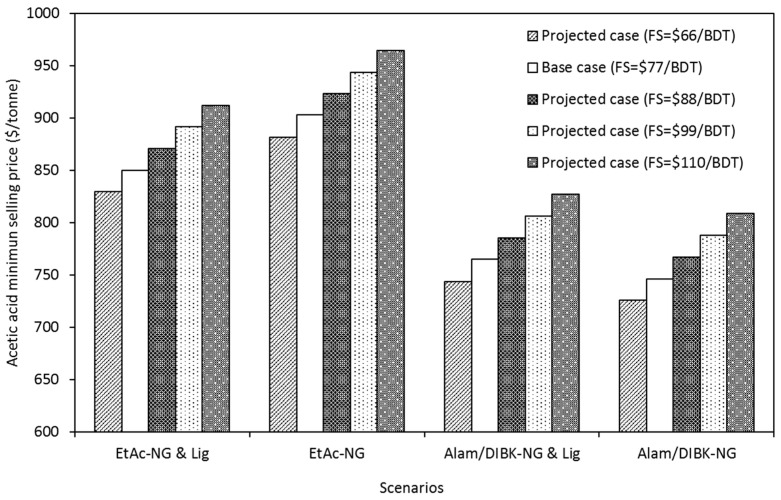
Effect of feedstock (FS) cost on the minimum selling price of acetic acid for different projected cases for a biorefinery with a capacity of 120,650 tons of acetic acid per year.

Comparing the results shown in Figure 3 with those in Table 7, it can be seen that while feedstock cost has a large effect on the minimum selling price, that effect has less impact when considered with the historical fluctuations in natural gas prices.

**Figure 4:**
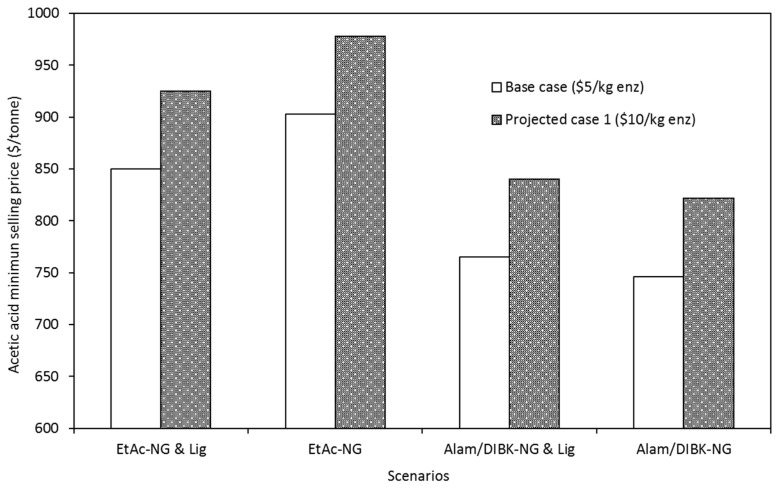
Effect of the enzyme (enz) cost on minimum selling price of acetic acid for a projected case (enz = 10 USD/kg) for a biorefinery with a capacity of 120, 650 tons of acetic acid per year.

The sensitivity of the acetic acid minimum selling price to enzyme cost was investigated by doubling the enzyme cost (10 USD/kg) from the base case (5 USD/kg). The results of the sensitivity analysis are shown in Figure 4 for all the scenarios. Doubling the enzyme cost increased the acetic acid minimum selling price by an average of USD 75/tons over all the scenarios. The effect of the enzyme cost was similar for all the cases because the enzyme dosage was identical for all the scenarios analyzed.

Comparing Table 7, Figure 3, and Figure 4, it can be seen that the biorefinery is most susceptible to variability in natural gas prices and least susceptible to enzyme costs for the scenarios investigated in this study. Feedstock cost was found to have an impact somewhere between those of natural gas prices and enzyme costs.

## 4. Conclusions

In this study, a comprehensive techno-economic analysis was performed to evaluate and compare the technical and economic feasibility of GAA production, starting from poplar biomass feedstock. ASPEN chemical engineering software was used to simulate acetic acid production at a bioconversion biorefinery that produces 120,650 tons per year of acid product. Separation of acetic acid from water is a major expense, and requires considerable energy, when producing pure acid starting with biomass. Consequently, recovery of the acid product using either ethyl acetate or alamine as organic solvents for liquid–liquid extraction purification was investigated. The alamine recovery process has less than one-half of the steam demand of the ethyl acetate recovery process, even though it uses an additional distillation column. As with most bioconversion processes, the calculated capital cost of the simulated biorefineries is high. The modeled facility, depending on how it is configured, costs from $197 million to $245 million. The configurations using alamine recovery were slightly less expensive, since they require a smaller boiler. Using a moderate pressure, natural gas-fired boiler instead of a high-pressure boiler firing a combination of lignin and natural gas saved approximately 18% in capital cost, but incurred an increased operating cost by requiring the purchase of electrical power to run the process.

Cash operating costs ranged from $441 to $618 per ton of acetic acid, with the alamine process using the lignin and natural gas-fired boiler having the lowest operating cost. Generating power onsite with the lignin/natural gas boiler reduced operating cost by avoiding purchase of electricity and providing modest revenue with the sale of excess electricity. The minimum acetic acid selling price to achieve a 15% internal rate of return ranged from $746 to $903 per ton of acetic acid, with the alamine process using a natural gas-fired boiler having the lowest minimum selling prices. These selling prices are higher than the current acetic acid selling price of $400/ton, but most of them fall within the historic range of the acetic acid selling price of $400 to $850 per ton. Sensitivity analysis shows that this process is especially sensitive to variation in natural gas prices because of the energy intensity of the recovery process and the historic volatility in natural gas pricing. This work provides new opportunities to the bioacetic acid market by establishing a feasible and sustainable approach to produce acetic acid from poplar biomass via fermentation using a high boiling point solvent for the purification process.

## Figures and Tables

**Table 1:** Parameters for discounted cash flow analysis.

Item	Parameter
Discount rates (nominal, compounded yearly)	15%
Project lifetime (plant operation)	20 years
Construction time	3 years
Equity share	40%
Tax rate	0%
Working capital (% of Fixed Capital Investment)	10%, returned at project completion

**Table 2:** Temperature and energy consumption for liquid–liquid extraction of acetic acid.

	Ethyl Acetate		Alamine/DIBK	
Process	Temp (°C)	Heat Duty (MJ/h)	Temp (°C)	Heat Duty (MJ/h)
Dehydration tower	117	343,000	167	110,000
Stripping tower 1	100	164,000	183	64,000
Stripping tower 2	N/A	N/A	168	10,000
Total	N/A	508,000	N/A	184,000

**Table 3:** Major biorefinery unit operation steam uses per kg of acetic acid produced.

	Ethyl Acetate		Alamine/DIBK	
Process	Steam Usage (kg)	Steam Temp (°C); Pressure (kPa)	Steam Usage (kg)	Steam Temp (°C); Pressure (kPa)
Pretreatment	1.4	275; 1320	1.4	275; 1320
Dehydration tower	10.7	126; 241	3.4	198; 621
Stripping tower 1	5.1	126; 241	2.0	198; 621
Stripping tower 2	N/A	N/A	0.3	198; 621

**Table 4:** Net biorefinery electricity per kg of acetic acid is produced.

	Ethyl Acetate		Alamine/DIBK	
Electricity (kWh)	Lignin and Natural Gas	Natural Gas	Lignin and Natural Gas	Natural Gas
Consumed	0.41	0.31	0.37	0.29
Generated	2.99	N/A	1.23	N/A
Excess generated	2.58	N/A	0.86	N/A

**Table 5:** Capital costs breakdown for a biorefinery with a capacity of 120,650 tons acetic acid/year.

	Ethyl Acetate		Alamine/DIBK	
Fixed Capital (million USD)	Lignin and Natural Gas	Natural Gas	Lignin and Natural Gas	Natural Gas
Feedstock through fermentation	119	119	119	119
Acetic acid separation/purification	10.9	10.9	11.5	11.5
Steam plant	79.7	31.9	57.5	20.7
Wastewater treatment	35.4	35.4	35.4	35.4
Total	245	197	223	186

**Table 6:** Cash operating cost and minimum selling price for a biorefinery with a capacity of 120,650 tons acetic of acid/year.

	Ethyl Acetate		Alamine/DIBK	
Operating Cost (USD/Ton of Acetic Acid)	Lignin and Natural Gas	Natural Gas	Lignin and Natural Gas	Natural Gas
Feedstock	144	144	144	144
Cellulase	83	83	83	83
Other raw materials *	46	40	50	42
Waste disposal	3	0	3	0
Natural gas (0.004 USD/MJ)	219	262	79	122
Electricity (0.05 USD/MJ)	−134	17	−45	15
Lignin as fuel (81 USD/ton)	N/A	−43	N/A	−43
Fixed manufacturing cost	134	115	127	111
Total cash cost	495	618	441	474
Minimum selling price	850	903	765	746

* Other raw materials: H_2_SO_4_, solvents, ammonia, fermentation nutrients, etc.

**Table 7:** The minimum selling price of acetic acid for base case scenario (natural gas = 4.2 USD/GJ) and two projected cases (natural gas = 12.7 USD/GJ and 2.4 USD/GJ) for a biorefinery with a capacity of 120,650 tons/year.

	Ethyl Acetate		Alamine/DIBK	
Minimum Selling Price (USD/ton of acetic acid)	Lignin and Natural Gas	Natural Gas	Lignin and Natural Gas	Natural Gas
Base case (natural gas = $4.2/GJ)	850	903	765	746
Projected case 1 (natural gas = $2.4/GJ)	755	789	730	694
Projected case 2 (natural gas = $12.7/GJ)	1,265	1,399	915	978
